# Peroxynitrite supports a metabolic reprogramming in merlin-deficient Schwann cells and promotes cell survival

**DOI:** 10.1074/jbc.RA118.007152

**Published:** 2019-06-06

**Authors:** Jeanine C. Pestoni, Stephani Klingeman Plati, Oliver D. Valdivia Camacho, Marisa A. Fuse, Maria Onatunde, Nicklaus A. Sparrow, Matthias A. Karajannis, Cristina Fernández-Valle, Maria Clara Franco

**Affiliations:** ‡ Department of Biochemistry and Biophysics, College of Science, Oregon State University, Corvallis, Oregon 97331; § Burnett School of Biomedical Sciences, College of Medicine, University of Central Florida, Orlando, Florida 32827; ¶ Department of Pediatrics and Otolaryngology, NYU Langone Health, New York, New York 10016

**Keywords:** Schwann cells, redox signaling, bioenergetics, energy metabolism, tumor metabolism, merlin, neurofibromatosis, nitration, peroxynitrite, schwannoma

## Abstract

Neurofibromatosis type 2 (NF2) is an autosomal-dominant disorder characterized by the development of bilateral vestibular schwannomas. The *NF2* gene encodes the tumor suppressor merlin, and loss of merlin activity promotes tumorigenesis and causes NF2. Cellular redox signaling has been implicated in different stages of tumor development. Among reactive nitrogen species, peroxynitrite is the most powerful oxidant produced by cells. We recently showed that peroxynitrite-mediated tyrosine nitration down-regulates mitochondrial metabolism in tumor cells. However, whether peroxynitrite supports a metabolic shift that could be exploited for therapeutic development is unknown. Here, we show that vestibular schwannomas from NF2 patients and human, merlin-deficient (MD) Schwann cells have high levels of endogenous tyrosine nitration, indicating production of peroxynitrite. Furthermore, scavenging or inhibiting peroxynitrite formation significantly and selectively decreased survival of human and mouse MD-Schwann cells. Using multiple complementary methods, we also found that merlin deficiency leads to a reprogramming of energy metabolism characterized by a peroxynitrite-dependent decrease of oxidative phosphorylation and increased glycolysis and glutaminolysis. In MD-Schwann cells, scavenging of peroxynitrite increased mitochondrial oxygen consumption and membrane potential, mediated by the up-regulation of the levels and activity of mitochondrial complex IV. This increase in mitochondrial activity correlated with a decrease in the glycolytic rate and glutamine dependence. This is the first demonstration of a peroxynitrite-dependent reprogramming of energy metabolism in tumor cells. Oxidized proteins constitute a novel target for therapeutic development not only for the treatment of NF2 schwannomas but also other tumors in which peroxynitrite plays a regulatory role.

## Introduction

Mutations in the *NF2*^4^ gene coding for the tumor suppressor merlin cause neurofibromatosis type 2 (NF2), an autosomal-dominant tumor predisposition disorder of the nervous system (1, 2). Individuals with NF2 develop multiple benign tumors including schwannomas, meningiomas, and ependymomas throughout their lifetime (3). Merlin controls cell size, morphology, proliferation, and survival through the regulation of multiple cell signaling pathways (4–6). These include mitogenic receptors (EGFR, erbB2, and CD44), the PI3K/Akt/mTOR pathway, extracellular matrix receptors (β1 integrins), RasGTPase, and the MST-YAP pathway that controls organ development and homeostasis (4, 7–13). Increasing cell density converts merlin from a growth-permissive to a growth-inhibitory conformation (14). Because merlin plays such a key regulatory role in Schwann cell proliferation and survival, loss of merlin function promotes tumorigenesis (14, 15).

Redox signaling and oxidative stress have been implicated in different stages of tumor development (16, 17). Reactive oxygen and nitrogen species have the mutagenic potential needed in the first steps of tumorigenesis. However, reactive nitrogen and oxygen species can also regulate signal transduction pathways favoring proliferation, survival, and promoting cell motility and angiogenesis, which facilitates metastatic processes in cancer (16). Among the reactive nitrogen species, peroxynitrite is a powerful oxidant formed by the diffusion-limited reaction of nitric oxide (NO) and superoxide (O_2_^˙̄^) (18). The active products of peroxynitrite decomposition react with tyrosine residues in proteins forming nitrotyrosine (19). Tyrosine nitration is found in several pathologies, including cancer, neurodegeneration, and inflammation (19–24). Although formation of peroxynitrite and tyrosine nitration is associated with induction of cell death in neurodegenerative conditions, it is also detected in malignant gliomas and colon cancer as well as in invasive breast carcinoma, where it correlates with lymph node infiltration and poor prognosis (25–27).

We showed that nitration of the molecular chaperone heat shock protein 90 (Hsp90) on one of two specific residues, tyrosine 33 and 56, switches the molecular chaperone from a prosurvival protein to mediator of motor neuron death through a toxic gain-of-function (28). However, in tumor cells nitration of Hsp90 on tyrosine 33 but not 56 down-regulates mitochondrial activity through the formation of a protein complex, suggesting that differential nitration states of Hsp90 regulate distinct aspects of cell metabolism (29). These observations provide strong evidence for a role of peroxynitrite in regulating key molecular processes involved in pathology.

Here we show that peroxynitrite is necessary for NF2 schwannoma cell survival. Prevention of tyrosine nitration decreased cell survival of mouse and human merlin-deficient (MD) but not wildtype (WT) Schwann cells. Furthermore, we show for the first time that peroxynitrite controls energy metabolism of human MD-Schwann cells through the down-regulation of mitochondrial activity and increasing glycolysis and glutamine dependence, a metabolic phenotype shared by other tumor cell types. We observed a significant decrease in the levels and activity of the mitochondrial oxidative phosphorylation complexes in the absence of merlin expression, leading to a decrease in mitochondrial oxygen consumption rate (OCR). Scavenging peroxynitrite-derived radicals with urate reverted the metabolic phenotype of human MD-Schwann cells back to that of isogenic WT-Schwann cells at least in part by increasing the levels and activity of the cytochrome *c* oxidase (complex IV). Together these observations reveal that peroxynitrite plays an important role in the regulation of the metabolic phenotype of NF2 schwannoma cells. Proteins oxidized by peroxynitrite could be exceptional targets for the development of tumor-directed therapies for the treatment of NF2 and possibly for treatment of other solid tumors.

## Results

### Loss of merlin expression leads to increased peroxynitrite production in Schwann cells

**Figure 1.**
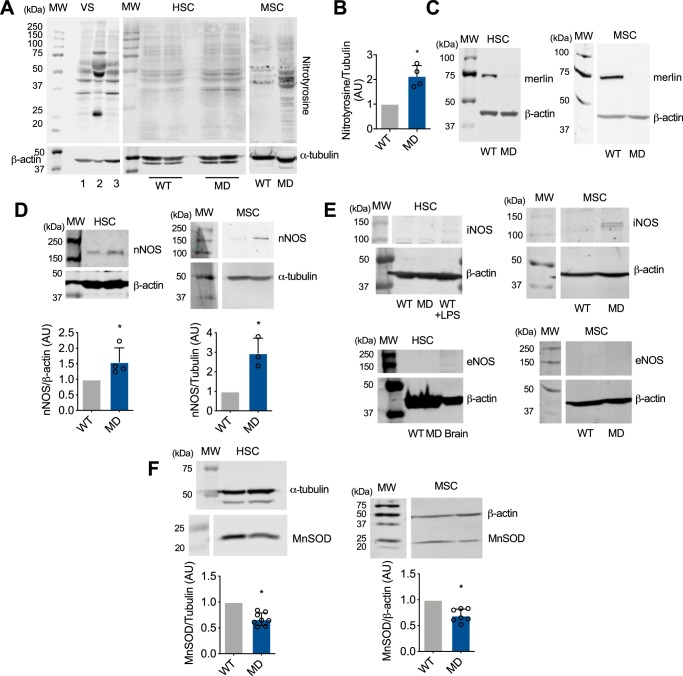
**Tyrosine nitration is increased in NF2 schwannoma cells.** *A,* representative IR Western blots showing nitrotyrosine staining of vestibular schwannoma samples from three NF2 patients (*VS 1 to 3*), and human (I) and mouse (*MSC*) WT- and MD-Schwann cells. α-Tubulin and β-actin were used as loading controls. *B,* quantitation of nitrotyrosine levels in HSC (*n* = 4). *C,* loss of merlin expression in both human and mouse MD-Schwann confirmed by IR Western blotting. *D–F*, representative Western blots showing the levels of: *D,* nNOS (*n* = 3–4); *E,* inducible and endothelial NOS (iNOS and eNOS, respectively, *n* = 4–5); and *F,* MnSOD (*n* = 7–8) in human and mouse WT- and MD-Schwann cells. Homogenate from human WT-Schwann cells treated with 1 μm lipopolysaccharide (*LPS*) for 24 h was used as positive control for iNOS expression, and brain homogenate as control for nNOS and eNOS expression. *Columns* represent the mean ± S.D. of the respective Western blotting band quantitation normalized against α-tubulin or β-actin and expressed relative to WT-Schwann cells. *, *p* < 0.05 *versus* WT by either Student's *t* test or Mann-Whitney test.

Tyrosine nitration, a marker of peroxynitrite formation, is present in several tumor cell types. In cancer, expression of inducible nitric-oxide synthase and high nitration levels correlate with metastasis and poor prognosis (25–27, 30–33), suggesting that peroxynitrite may regulate key processes in tumor cells. Peroxynitrite production was investigated in vestibular schwannomas (VS) from NF2 patients, human and mouse wildtype (WT) Schwann cells, as well as in human and mouse Schwann cells deficient in merlin expression either by merlin knockdown, or by merlin knockout as a result of NF2 exon 2 deletion (34, 35). Protein tyrosine nitration was found in samples from three vestibular schwannomas from NF2 patients (Fig. 1*A*). Increased tyrosine nitration and a similar pattern of nitrated proteins was observed in both human and mouse MD-Schwann cells (Fig. 1, *A* and *B*), with undetectable levels of merlin (Fig. 1*C*). To determine whether the increase in tyrosine nitration observed in the absence of merlin expression was due to an increase in nitric oxide production, the expression levels of the three isoforms of nitric-oxide synthase, neuronal (nNOS), inducible (iNOS), and endothelial (eNOS), was assessed by quantitative IR Western blotting. There was a significant increase in the expression of nNOS in human and mouse MD-Schwann cells compared with WT-Schwann cells (Fig. 1*D*). In addition to higher levels of nNOS, expression of iNOS was observed in mouse MD-Schwann cells but not WT-Schwann cells (Fig. 1*E*), whereas expression of eNOS was not detected in any of the cell types (Fig. 1*E*). The increase in NOS expression correlated with a decrease in the expression of the mitochondrial antioxidant enzyme manganese superoxide dismutase (MnSOD) (Fig. 1*F*), suggesting that the loss of merlin expression induces an increase in peroxynitrite formation by increasing nitric oxide production while decreasing superoxide clearance by MnSOD in NF2 schwannoma cells.

### Scavenging of peroxynitrite-derived radicals decreases cell survival in Schwann cells deficient in merlin expression

**Figure 2.**
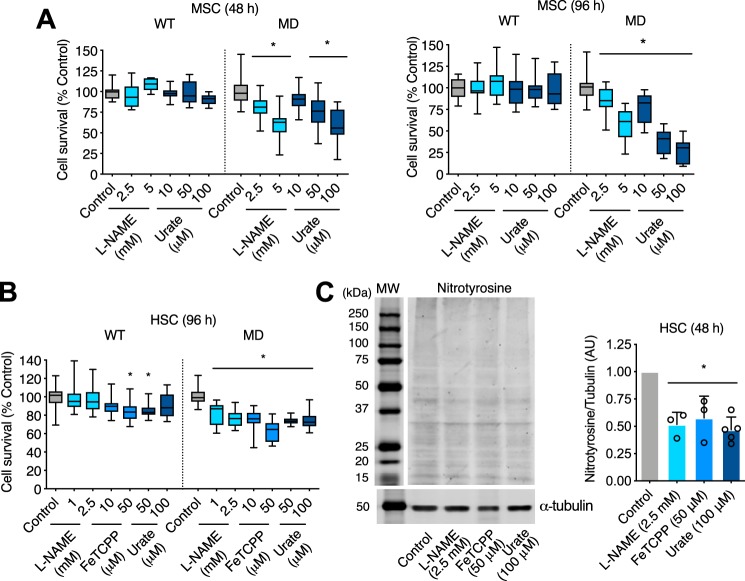
**Peroxynitrite selectively regulates human and mouse MD-Schwann cell survival.** *A* and *B,* survival of: mouse (*A*) and human WT- and MD-Schwann cells (*B*) was assessed by nuclear staining with crystal violet, as described under “Experimental procedures,” after 48 and 96 h of incubation in the absence and presence of the NOS inhibitor l-NAME (1, 2.5, and 5 mm), the superoxide and peroxynitrite scavenger FeTCPP (10 and 50 μm), and urate (10, 50, and 100 μm), a natural scavenger of peroxynitrite-derived radicals. Cell survival is expressed as percentage of untreated control (*n* = 6–7 with 8 replicates). *C,* representative IR Western blotting for nitrotyrosine of MD-Schwann cells incubated in the absence and presence of l-NAME (2.5 mm), FeTCPP (50 μm), and urate (100 μm) for 48 h. On the *right*, quantitation of the corresponding bands normalized against α-tubulin. *Columns* represent the mean ± S.D. (*n* = 3–5) expressed relative to untreated control. *, *p* < 0.01 *versus* untreated control by Kruskal Wallis test followed by Dunn's post test.

To determine whether peroxynitrite played a relevant role in tumor cell survival in NF2 schwannoma cells, we prevented endogenous production of peroxynitrite by inhibiting NOS activity with L-NAME, scavenging superoxide and peroxynitrite using the iron porphyrin FeTCPP (36), and by incubating the cells with urate to directly scavenge peroxynitrite-derived radicals. Although uric acid, the end product of purine metabolism, reacts with peroxynitrite relatively slowly, it is a very effective natural scavenger of the radical products of peroxynitrite decomposition, responsible for peroxynitrite downstream signaling (19, 37–39). At physiological pH, uric acid exists mainly as urate. Urate prevents peroxynitrite-mediated toxicity *in vitro* and *in vivo*, and it has been shown to be neuroprotective in neurodegenerative conditions (37, 40). At physiological pH, urate also efficiently and selectively repairs tyrosyl radicals, preventing tyrosine nitration (41). Furthermore, micromolar concentrations of urate can efficiently compete *in vivo* to inhibit tyrosine nitration (41–44). Inhibition of NO production, and scavenging of peroxynitrite-derived radicals with urate for 48 and up to 96 h dramatically decreased mouse MD-Schwann cell viability without affecting survival of mouse WT-Schwann cells (Fig. 2*A*). A significant decrease in cell survival was also observed in human MD-Schwann cells after 96 h of treatment with l-NAME, FeTCPP, and urate compared with human WT-Schwann cells (Fig. 2*B*), suggesting that peroxynitrite selectively supports survival of NF2 schwannoma cells. Treatment with l-NAME, FeTCPP, and urate significantly reduced the levels of tyrosine nitration of human MD-Schwann cells after 48 h incubation, confirming scavenging of peroxynitrite-derived radicals (Fig. 2*C*).

### Peroxynitrite decreases the mitochondrial activity of human MD-Schwann cells

**Figure 3.**
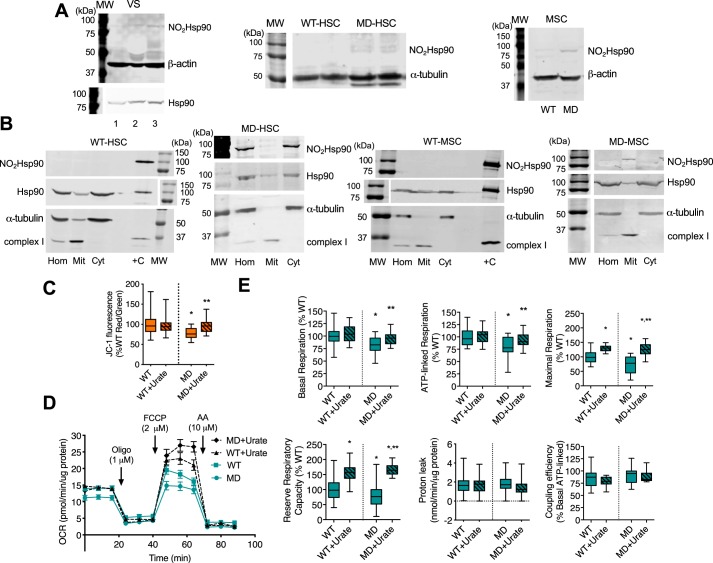
**Peroxynitrite selectively down-regulates mitochondrial metabolism in human MD-Schwann cells.** *A,* representative IR Western blots of nitrated and total Hsp90 in homogenates of vestibular schwannomas from three NF2 patients (*VS 1 to 3*), and human (*HSC*) and mouse (*MSC*) WT- and MD-Schwann cells (*n* = 3–4); and *B,* in homogenates (*Hom*) and mitochondrial (*Mit*) and cytosolic (*Cyt*) fractions of human and mouse WT- and MD-Schwann cells subjected to subcellular fractionation (*n* = 3). +*C*: positive control, nitrated recombinant Hsp90. *C,* the mitochondrial membrane potential of human WT- and MD-Schwan cells after 48 h incubation in the absence or presence of urate (100 μm) was determined after incubation of the cells with the probe JC-1 (5 μm) for 30 min and expressed as the percentage of the ratio of *red* over *green* JC-1 fluorescence (*n* = 3 with 8 replicates). *, *p* < 0.05 *versus* untreated WT; **, *versus* untreated MD by Kruskal-Wallis test. *D,* the OCR of human WT- and MD-Schwann cells after 48 h of incubation in the absence and presence of urate (100 μm) was measured by extracellular flux analysis in basal conditions and after the sequential addition of oligomycin (1 μm, oligo), FCCP (2 μm), and antimycin A (10 μm, *AA*) (mean ± S.E. of 5 independent experiments with 5 replicates). *E,* analysis of the different OCR parameters after sequential addition of the inhibitors, expressed as percentage of WT-Schwann cells (*n* = 5 with 5 replicates). *, *p* < 0.001 *versus* untreated WT-Schwann cells; **, *versus* untreated MD-Schwann cells by one-way ANOVA followed by Bonferroni post test.

We have shown that site-specific nitration of the chaperone Hsp90 regulates different aspects of cell metabolism (28, 29). Nitrated Hsp90 associates with mitochondria and down-regulates mitochondrial activity, a hallmark of tumor cell energy metabolism (29). Because we observed an increase in tyrosine nitration in vestibular schwannomas from NF2 patients and in human and mouse MD-Schwann cells, we investigated whether Hsp90 was endogenously nitrated in these cells and in tumor samples. We found nitrated Hsp90 in vestibular schwannomas and in human and mouse MD-Schwann cells (Fig. 3*A*). Moreover, nitrated Hsp90 associated with mitochondria in both human and mouse MD-Schwann cells (Fig. 3*B*). Importantly, nitrated Hsp90 was not detected in WT-Schwann cells in any condition tested (Fig. 3, *A* and *B*). In agreement with the mitochondrial localization of nitrated Hsp90, human MD-Schwann cells exhibited a significantly lower mitochondrial membrane potential compared with isogenic WT-Schwann cells, as determined by measuring the ratiometric JC-1 fluorescence signal. To evaluate whether peroxynitrite was responsible for the decrease observed in mitochondrial membrane potential in human MD-Schwann cells, we scavenged peroxynitrite-derived radicals in human WT- and MD-Schwann cells by incubation with urate for 48 h. Urate treatment reversed the decrease observed in the mitochondrial membrane potential of MD-Schwann cells to WT levels (Fig. 3*C*), suggesting that peroxynitrite may be responsible for the down-regulation of mitochondrial activity in human MD-Schwann cells. In agreement with this observation, human MD-Schwann cells showed decreased basal and ATP-linked OCR, and maximal respiration and reserve capacity, compared with WT-Schwann cells without changes in proton leak or mitochondrial coupling (Fig. 3, *D* and *E*). Scavenging of peroxynitrite-derived radicals had a significant and selective effect on several aspects of mitochondrial metabolism in MD-Schwann cells. Urate treatment increased mitochondrial basal and ATP-linked respiration to levels comparable with those found in WT-Schwann cells, whereas the maximal respiration and reserve respiratory capacity were increased to levels above those of WT-Schwann cells (Fig. 3, *D* and *E*). Treatment of the cells with urate did not affect the proton leak or the mitochondrial coupling efficiency (Fig. 3, *D* and *E*). We also observed an increase in maximal respiration and reserve respiratory capacity in human WT-Schwann cells after urate treatment (Fig. 3, *D* and *E*), suggesting that low levels of peroxynitrite formation may regulate Schwann cell mitochondrial metabolism only under stress conditions, which may explain the slight decrease in survival observed in these cells after incubation with FeTCPP and urate (Fig. 2).

### Peroxynitrite decreases the levels of mitochondrial oxidative phosphorylation in human MD-Schwann cells

**Figure 4.**
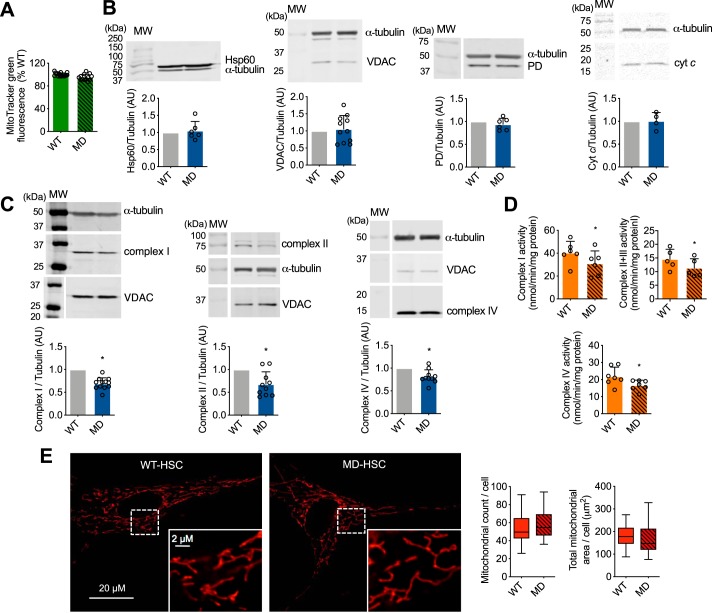
**Loss of merlin expression in human Schwann cells leads to decreased mitochondrial oxidative phosphorylation complex levels and activity.** *A,* the mitochondrial content of human WT- and MD-Schwann cells was determined after 30 min incubation with the mitochondrial membrane potential-independent probe MitoTracker Green (200 nm) and expressed as percentage of WT-Schwann cells (*n* = 3 with 4 replicates). *B* and *C,* representative IR Western blots of mitochondrial proteins in human WT- and MD-Schwann cells: *B,* Hsp60, VDAC, PD, and cytochrome *c* (cyt *c*); and *C,* complex I (NDUFA9 subunit), complex II (SDHA subunit), and complex IV (subunit COX IV). *Below*, quantitation of the corresponding bands normalized against α-tubulin and expressed relative to WT-Schwann cells (*n* = 4–12). *D,* mitochondrial complex I, II + III, and IV activities were assessed in disrupted isolated mitochondria of WT- and MD-Schwann cells. Complex I activity was measured at 340 nm by the rotenone-sensitive reduction of ubiquinone-1 in the presence of potassium cyanide and NADH. Complex II + III activity was determined by the antimycin A-sensitive reduction of cytochrome *c* at 550 nm in the presence of potassium cyanide and succinate. Complex IV activity was determined by monitoring the potassium cyanide-sensitive oxidation of cytochrome *c* at 550 nm. Activities are expressed as nanomole/min/mg of protein; *columns* represent the mean ± S.D. (*n* = 6–7). *E,* representative images of human WT- and MD-Schwann cell mitochondrial content in live cells after 30 min incubation with MitoTracker Red-FM (350 nm). At the *bottom*, higher magnification of the indicated field (in *white*) is shown. On the *right*, mitochondrial count and total mitochondrial area per cell (*n* = 4 with 6 replicates). *, *p* < 0.05 *versus* WT- Schwann cells by Student's *t* test.

The changes in mitochondrial activity observed in MD-Schwann cells could be due to a decrease in mitochondrial content rather than mitochondrial function. Thus, we determined the mitochondrial content using two different approaches: 1) incubating the cells in the presence of the mitochondrial membrane potential-independent probe MitoTracker Green, and 2) by assessing the levels of several mitochondrial proteins by Western blotting. No differences were detected between human MD- and WT-Schwann cells in the MitoTracker Green fluorescence signal, suggesting that there were no changes in mitochondrial content due to loss of merlin function (Fig. 4*A*). We corroborated this observation by quantitative IR Western blot analysis of several mitochondrial proteins with different submitochondrial locations, including voltage-dependent anion channel (VDAC) of the outer membrane, cytochrome *c* located in the intermembrane space, pyruvate dehydrogenase and heat shock protein 60 (Hsp60) located in the matrix, and components of the oxidative phosphorylation (complex I, II, and IV subunits) embedded in the inner membrane. There were no changes in the expression of VDAC, cytochrome *c*, pyruvate dehydrogenase, and Hsp60 between WT- and MD-Schwann cells (Fig. 4*B*), further supporting the results using MitoTracker Green. Surprisingly, there was a significant decrease in the levels of subunits of complex I (NADH:ubiquinone oxidoreductase, NDUFA9 subunit), complex II (succinate:ubiquinone oxidoreductase, SDHA subunit), and complex IV (cytochrome *c* oxidase, COX IV subunit) in human MD, compared with WT-Schwann cells (Fig. 4*C*). The decrease in oxidative phosphorylation complex levels correlated with a significant decrease in complex I, II + III, and IV activity in human MD-Schwann cells (Fig. 4*D*), without changes in either mitochondrial count or total mitochondrial area per cell (Fig. 4*E*). Together these results imply that upon loss of merlin function, there is a reprogramming of energy metabolism in human MD-Schwann cells through a decrease in mitochondrial respiratory chain levels and activity.

**Figure 5.**
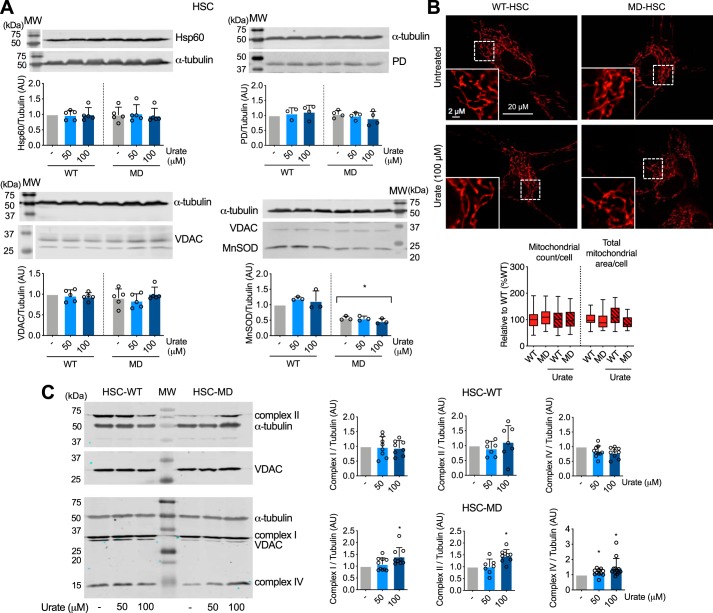
**Scavenging of peroxynitrite-derived radicals reverses the decrease of mitochondrial complex levels in human MD-Schwann cells.** Human WT- and MD-Schwann cells were cultured in the absence and presence of urate (50 and 100 μm) for 48 h before performing IR Western blots. *A,* representative IR Western blots of mitochondrial proteins Hsp60, PD, VDAC, and MnSOD in human WT- and MD-Schwann cells. *Below*, quantitation of the corresponding bands normalized against α-tubulin and expressed relative to untreated WT-Schwann cells (*n* = 3–5). VDAC was used as a mitochondrial loading control in the blot corresponding to MnSOD. *B,* representative images of human WT- and MD-Schwann cell mitochondrial morphology after 48 h incubation in the absence and presence of urate (100 μm). On the *right*, mitochondrial count and total mitochondrial area per cell are expressed as percentage of WT-Schwann cells (*n* = 4 with 5–6 replicates). *C,* representative IR Western blots of complex I, II, and IV in cell homogenates from human WT- (*HSC-WT*) and MD-Schwann cells (*HSC-MD*). On the *right*, quantitation of the corresponding bands normalized against α-tubulin and expressed relative to untreated control (*n* = 7–12). VDAC was used as a mitochondrial loading control. *Columns* represent the mean ± S.D. *, *p* < 0.05 *versus* untreated WT- or MD-Schwann cells by Kruskal-Wallis test followed by Dunn's post test.

**Figure 6.**
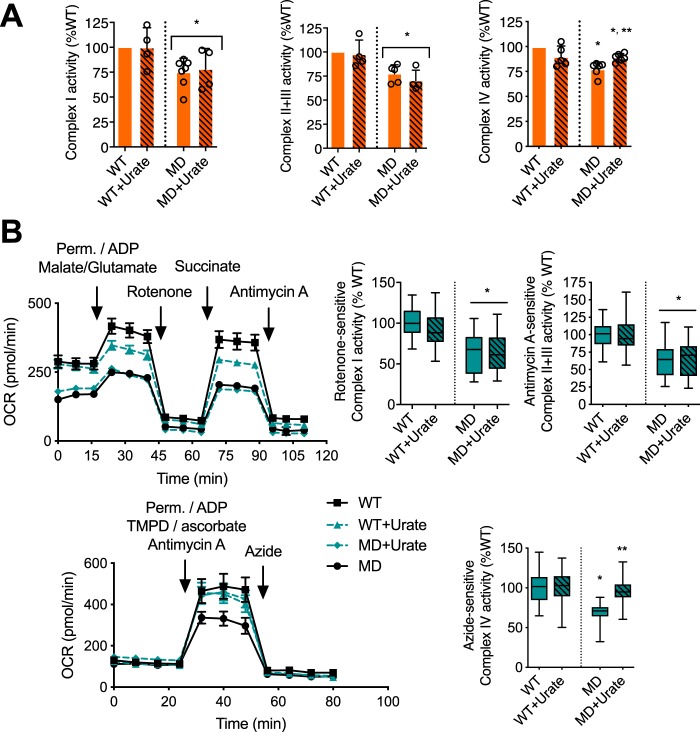
**Scavenging peroxynitrite-derived radicals reverses the decrease of mitochondrial complex IV activity in human MD-Schwann cells.** *A,* mitochondrial complex I, II + III, and IV activities were assessed in disrupted mitochondria of WT- and MD-Schwann cells isolated after 48 h incubation in the absence and presence of urate (100 μm). Complex activities are expressed as % inhibitor-sensitive complex activity respect to WT-Schwann cells. *Columns* represent the mean ± S.D. (*n* = 4–6). *B,* mitochondrial complex I, II + III, and IV activity was assessed by extracellular flux analysis in permeabilized mitochondria. On the *left*, representative OCR experiments (mean ± S.D. of 5 replicates). Cells were permeabilized during the first injection (*Perm*), and ADP was added to couple the activity of the complexes to ATP production. Malate-glutamate was added as substrate for complex I, succinate as substrate for complex II, and TMPD/ascorbate as substrate of complex IV. Azide was used to inhibit complex IV activity. Complex activities are expressed as % inhibitor-sensitive complex activity in respect to WT-Schwann cells (*n* = 3–4 with 5 replicates). *, *p* < 0.01 *versus* untreated WT- or MD-Schwann cells, and **, *p* < 0.01 *versus* untreated MD-Schwann cells by Kruskal-Wallis test followed by Dunn's post test.

To determine whether peroxynitrite played a role in the regulation of oxidative phosphorylation complex levels in human MD-Schwann cells, human WT- and MD-Schwann cells were cultured in the presence and absence of urate for 48 h to scavenge peroxynitrite-derived radicals before assessing the levels of mitochondrial proteins. Treatment of human WT- or MD-Schwann cells with urate did not affect the levels of mitochondrial proteins such as Hsp60, PD, VDAC, and MnSOD (Fig. 5*A*), nor the mitochondrial content (Fig. 5*B*). As expected, upon urate treatment MnSOD levels remained significantly reduced in human MD-Schwann cells compared with isogenic WT-Schwann cells (Fig. 5*A*). In contrast, incubation with urate significantly increased the levels of mitochondrial complex I, II, and IV in MD-Schwann cells, without affecting complex levels in human WT-Schwann cells (Fig. 5*C*). The increase in mitochondrial complex levels in human MD-Schwann cells was accompanied by a significant increase in complex IV activity in disrupted isolated mitochondria, without changes in the activity of complex I and II + III (Fig. 6*A*). Furthermore, the decrease in complex I, II + III, and IV activities in human MD-Schwann cells compared with WT-Schwann cells was more pronounced when measured in permeabilized cells with intact mitochondrial network (Fig. 6*B*). In these conditions, scavenging of peroxynitrite completely reversed complex IV activity to WT-Schwann cell levels (Fig. 6*B*), suggesting that whereas additional mechanism(s) may regulate the activity of the oxidative phosphorylation in MD-Schwann cells, the regulation of complex IV activity by tyrosine nitration is a limiting step. Together these observations suggest that peroxynitrite regulates a shift of energy metabolism in human MD-Schwann cells.

### Peroxynitrite induces a metabolic shift to glycolysis and glutaminolysis in human MD-Schwann cells

**Figure 7.**
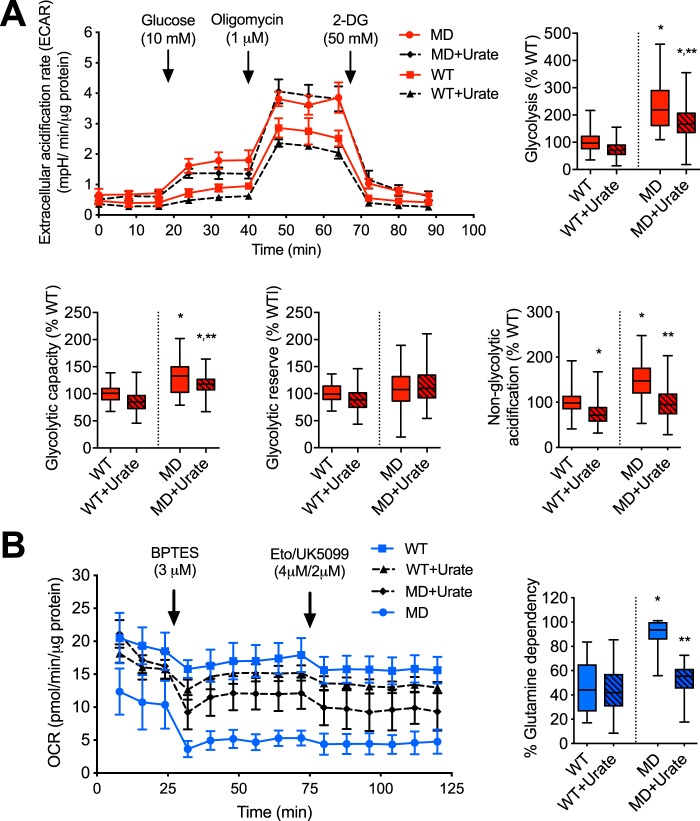
**Peroxynitrite supports a metabolic shift toward glycolysis and glutamine dependence in human MD-Schwann cells.** *A,* human WT- and MD-Schwann cells were cultured in the absence and presence of urate (100 μm) for 48 h before measuring the extracellular acidification rate (ECAR). Glutamine (1 mm) was added to the culture media followed by the sequential addition of glucose (10 mm), oligomycin (1 μm), and the glycolysis inhibitor 2-deoxyglucose (*2-DG*), as shown in the representative experiment on the *left* (mean ± S.D. of 5 replicates). On the *right*, analysis of the different parameters after the addition of substrate and inhibitors, expressed as percentage of WT-Schwann cells (*n* = 4 with 5 replicates). *, *p* < 0.01 *versus* untreated WT-Schwann cells; **, *versus* untreated MD-Schwann cells by Kruskal-Wallis test followed by Dunn's post test. *B,* the percentage of glutamine dependence of human WT- and MD-Schwann cells incubated in the absence and presence of urate for 48 h was determined by assessing the OCR in basal conditions and after the sequential addition of BPTES (3 μm), and Etoximar (4 μm) and UK5099 (2 μm), as shown in the representative experiment on the *left* (mean ± S.D. of 5 replicates). On the *right*, glutamine dependence expressed as percentage of WT-Schwann cells (*n* = 4 with 5 replicates) is shown. *, *p* < 0.001 *versus* untreated WT-Schwann cells; **, *versus* untreated MD-Schwann cells by one-way ANOVA followed by a Bonferroni post test.

**Figure 8.**
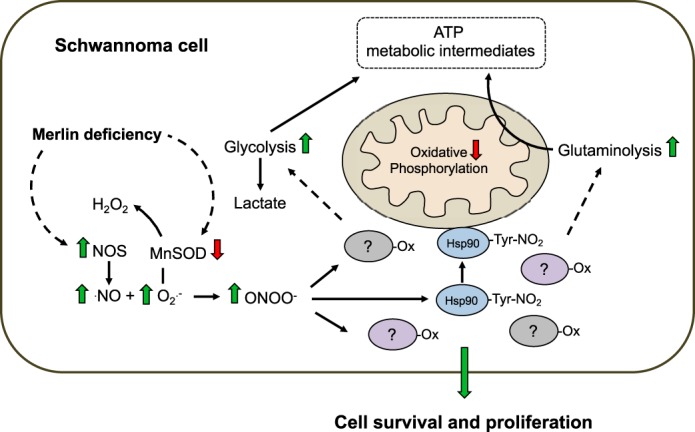
**Peroxynitrite supports a metabolic reprogramming in human NF2-associated schwannoma cells.** Peroxynitrite supports a metabolic shift in human MD-Schwann cells characterized by decreased oxidative phosphorylation and increased glycolysis and glutamine dependence. *Dotted lines* indicate mechanisms yet to be elucidated. *Ox,* oxidized protein; -*Tyr-NO_2_*, nitrotyrosine.

In tumor cells, decreased mitochondrial activity correlates with increased aerobic glycolysis and/or glutaminolysis to produce ATP and the biosynthetic intermediates needed for cell proliferation (45, 46). We assessed the glycolytic rate of human WT- and MD-Schwann cells by extracellular flux analysis after incubation of the cells in the presence and absence of urate for 48 h. After addition of glucose to the culture media, human MD-Schwann cells showed increased glycolysis and increased glycolytic capacity, measured as the glycolytic rate in the presence of 1 μm oligomycin, compared with human WT-Schwann cells; both parameters were partially reversed after scavenging of peroxynitrite-derived radicals (Fig. 7*A*). In agreement with the increased reserved respiratory capacity observed in human WT-Schwann cells after urate treatment (Fig. 3*E*), there was a corresponding decrease in the glycolytic reserve of these cells (Fig. 7*A*), supporting a possible role for low levels of peroxynitrite formation in the regulation of energy metabolism in human WT-Schwann cells only under stress conditions. Notably, there was a significantly increased acidification of the culture medium by human MD-Schwann cells in the presence of glutamine as the sole fuel source (nonglycolytic acidification) that was completely reversed after incubation with urate. This suggests that peroxynitrite may favor a shift toward glutaminolysis in human MD-Schwann cells. Supporting these observations, human MD-Schwann cells showed increased glutamine dependence, measured as the capacity of the mitochondria to utilize alternative fuel sources (glucose and fatty acids) in the presence of BPTES, an inhibitor of glutaminase, the enzyme that converts glutamine to glutamate (Fig. 7*B*). Together, these results suggest that peroxynitrite supports a metabolic shift from mitochondrial oxidative phosphorylation toward glycolysis and glutaminolysis in human MD-Schwann cells (Fig. 8).

## Discussion

Until now, the metabolic profile of human NF2 schwannoma cells had been largely unknown. We provide the first demonstration that these cells have a distinct metabolic phenotype that set them apart from normal Schwann cells. Tumor cell metabolic reprogramming is increasingly under investigation as a source for novel therapeutic targets to selectively inhibit tumor cell growth and survival (47). Furthermore, we show that the metabolic reprogramming of NF2 schwannoma cells is tightly regulated by peroxynitrite, suggesting that novel therapeutic targets for NF2 treatment could be identified among oxidized proteins.

Redox signaling and oxidative stress leading to chronic inflammation have been linked to tumor development (16, 17). In this context, high expression of NOS and tyrosine nitration correlates with tumor growth and aggressiveness (30–33). Moreover, recent evidence suggests that the nerve microenvironment and an unresolved inflammatory response could play an important role in the development of NF2 schwannomas (48). We found that loss of merlin expression leads to increased levels of nNOS in mouse and human Schwann cells. In addition, mouse MD- but not WT-Schwann cells also expressed detectable levels of iNOS. Expression of NOS regardless of the isoform was associated with production of peroxynitrite, as evidenced by increased tyrosine nitration, which was beneficial for NF2 schwannoma cell survival. Interestingly, we also observed a decrease in the levels of MnSOD in NF2 schwannoma cells, suggesting decreased capacity of these cells to clear superoxide, further supporting peroxynitrite production. This antioxidant enzyme has been described for many years as a tumor suppressor due to its role in negatively regulating redox signaling pathways related to cell proliferation (49, 50). Moreover, several tumor cell types exhibit low levels of MnSOD (50–52), further supporting the role of peroxynitrite in the redox signaling processes regulating NF2 schwannoma cell survival and growth. However, we were unable to directly measure superoxide and nitric oxide using a diversity of probes. In our experience, the diffusion-limited reaction of peroxynitrite formation precludes direct measurements by competing with the slower reaction of nitric oxide and superoxide with the respective probes. In addition to l-NAME and urate significantly decreasing tyrosine nitration and survival of mouse and human MD-Schwann cells, incubation of human MD-Schwann cells with FeTCPP selectively decreased cell survival. In contrast, mouse WT- and MD-Schwann cells were both highly sensitive to FeTCPP treatment. This high sensitivity of mouse Schwann cells is shared by other cell types. The cells become pale green after incubation with FeTCPP and other iron-containing porphyrins, indicating the reduction of the metal to Fe^2+^, probably at the expense of intracellular reducing agents, which could be the cause of the decreased cell viability.

Peroxynitrite and peroxynitrite-derived radicals react with amino acids in proteins introducing oxidative modifications. This is the case for tyrosine, cysteine, methionine, histidine, and tryptophan residues (53). Oxidation of critical residues can lead to protein inhibition or activation, or in some cases to a gain-of-function. In fact, the activity of some metabolic enzymes, including the mitochondrial complexes, can be inhibited by oxidation of critical cysteine residues (19). To date, a very small number of proteins involved in cell survival pathways that are oxidized by peroxynitrite have been detected in tumor cells. In the context of tyrosine nitration by peroxynitrite-derived radicals, nitration of the tumor suppressor p53 leads to its aggregation and loss of DNA binding *in vitro*. Nitrated p53 has been detected in gliomas *in vivo* (25). Additionally, nitration of c-Src tyrosine kinase increases both its activity and downstream binding to its substrate in human pancreatic ductal adenocarcinoma (54), whereas *in vitro* nitration of ERK1/2 leads to its autophosphorylation and activation (55). In this context, we were the first to show that a nitrated protein regulates mitochondrial metabolism in tumor cells. Nitration of the molecular chaperone Hsp90 down-regulates energy metabolism in tumor cells by forming a complex that partially inhibits cytochrome *c* oxidase activity (29). Nitrated Hsp90 was present in vestibular schwannoma samples from NF2 patients, and it was associated with mitochondria in human MD- and mouse MD-Schwann cells, whereas the nitrated chaperone was not detected in WT-Schwann cells. The perturbation of Schwann cell energy metabolism can have profound physiological and pathological effects. Schwann cell mitochondrial metabolism is essential to the myelination process (56). In addition, Schwann cell abnormal lipid metabolism and mitochondrial dysfunction is a common cause of peripheral neuropathy (57, 58), highlighting the importance of understanding the role that Schwann cell metabolic changes have on schwannoma development. A hallmark of solid tumor metabolism is the switch from oxidative phosphorylation to aerobic glycolysis, known as the Warburg effect (59). We found that human NF2 schwannoma cells show decreased levels and activity of the mitochondrial respiratory chain, with concomitant decrease of the oxygen consumption rate. This observation agrees with a recent report showing decreased levels of complex I, III, and IV and reduced oxidative phosphorylation in schwannoma samples from patients compared with histomorphologically normal brain samples (60).

Inactivation of complex IV has the potential to induce tumor progression. Silencing of the complex induces a metabolic shift and a transcriptional reprogramming that leads to invasive behavior in nontumorigenic skeletal myoblasts, and increased invasiveness in breast and esophageal cancer cell lines (61). Furthermore, we and others have shown that even small changes in mitochondrial activity can have a great impact in cell metabolism (29, 62, 63). Scavenging of peroxynitrite-derived radicals in human MD-deficient Schwann cells reprogrammed their mitochondrial metabolism to that of isogenic WT-Schwann cells. Incubation of human MD-Schwann cells with urate not only significantly increased the levels of complex IV, but also increased its activity and the mitochondrial oxygen consumption rate to WT levels. Although scavenging of peroxynitrite-derived radicals restored the levels of complex I and II, there were no associated changes in activity, suggesting that the activity of these complexes may be regulated by other mechanisms, for example, at the level of complex assembly. Nevertheless, the complete restoration of human MD-Schwann cell oxygen consumption rate to that of WT-Schwann cells after urate treatment suggests that complex IV is the critical regulatory step. Nitrated Hsp90 is known to decrease mitochondrial metabolism through inhibition of complex IV activity (29). The presence of nitrated Hsp90 in human MD-Schwann cell mitochondria could contribute to the decrease in mitochondrial activity. However, the mechanism by which nitrated Hsp90 regulates NF2 schwannoma metabolism requires further study. Additionally, tyrosine nitration of one or more complex subunits could lead to partial inactivation of the complex. Nevertheless, the correlation between impairment of complex IV activity and the peroxynitrite-mediated decrease in complex IV levels suggests that this is the principal mechanism of regulation in human MD-Schwann cells. These observations imply that in NF2 schwannoma cells complex IV activity and hence mitochondrial metabolism may be tightly regulated by peroxynitrite through the regulation of complex IV levels and activity.

A decrease in the activity of the mitochondrial oxidative phosphorylation often correlates with increased glycolysis in tumor cells (59). We observed an increased glycolytic rate in human MD-Schwann cells compared with human WT-Schwann cells that was partially reversed by prevention of tyrosine nitration. Notably, there was also a significant increase in the nonglycolytic acidification of the culture medium by human MD-Schwann cells incubated with glutamine as the sole fuel source that was completely prevented after incubation of the cells with urate, suggesting that production of peroxynitrite increases glutaminolysis. Glutamine fuels the mitochondrial tricarboxylic acid cycle in tumor cells not only to produce ATP but also to provide metabolic intermediates. The tricarboxylic acid cycle interconnects many different pathways to produce amino acids, fatty acids, and nucleotides needed to increase biomass and support cell proliferation (45, 46). In agreement with these observations, it was recently shown that the mouse merlin-deficient cell line FH912 has increased glutaminolysis and fatty acid synthesis (64). Here we show that human MD-Schwann cells display increased glycolysis and almost complete glutamine dependence. Furthermore, this metabolic shift is supported by peroxynitrite. The fact that this oxidant exerts a tight, selective regulation on the metabolic shift in human NF2 schwannoma cells provides promising molecular targets for drug development among the proteins oxidized by peroxynitrite. Identifying the oxidized proteins, and the critical residues in those proteins involved in the regulation of energy metabolism in NF2 schwannomas will provide exceptional new targets for the development of therapies directed specifically to tumor cells.

## Experimental procedures

### Vestibular schwannoma samples from NF2 patients

Fresh frozen vestibular schwannoma samples from NF2 patients were collected under an Institutional Review Board (IRB) approved protocol at NYU Langone Health. All experiments were conducted conforming to the ARRIVE guidelines.

### Generation of merlin-deficient human Schwann cells

Primary human Schwann cells were purchased from ScienCell Research Laboratories (Carlsbad, CA) and transduced with human NF2 gene-specific shRNA lentiviral particles (Sigma) according with the manufacturer's instructions. The merlin-deficient cells were then selected by the addition of 0.5 mg/ml of puromycin to the culture medium, as previously described (65). Assessment of merlin deficiency was performed by Western blotting.

### Human and mouse Schwann cell cultures

Human WT-Schwann cells were obtained from ScienCell Research Laboratories (catalog number 1700). These cells are not listed as a commonly misidentified cell line by the International Cell Line Authentication Committee. Human MD-Schwann cells were generated in-house from isogenic WT-Schwann cells using human *NF2* gene-specific shRNA lentiviral particles (66). Low passages of both human WT- and MD-Schwann cells were cultured on CellBIND dishes (Corning, Fisher Scientific, Hampton, NH) in ScienCell Schwann cell media and routinely tested for mycoplasma contamination (LookOut Mycoplasma PCR Detection Kit; Sigma). Mouse WT-Schwann cells were isolated from sciatic nerves as previously described (67), with modifications (35). All dissections were performed following protocols approved by the University of Central Florida (UCF) Institutional Animal Care and Use Committee (IACUC) and animals were maintained in UCF's Vivarium that is certified by the International Association for Assessment and Accreditation of Laboratory Animal Care (AAALAC). Sciatic nerves were dissected from postnatal day 2 FVB/NJ mice and dissociated with 1 mg/ml of collagenase type 2 (Worthington Biochemical, catalog number LS004176) followed by 0.25% trypsin (Thermo Fisher, Waltham, MA). Once the cells reached ∼90% confluence, the mouse Schwann cells were purified using trypsinization and an adapted laminin selection (68) and cultured on 200 μg/ml of poly-l-lysine (Millipore Sigma, catalog number P4832) and 25 μg/ml of laminin (ThermoFisher, catalog number 23017015)-coated dishes (Corning) in growth medium: Dulbecco's modified Eagle's medium:F-12 medium (Gibco/ThermoFisher, catalog number 10565018) with 1% N2 supplement (Invitrogen/ThermoFisher, catalog number 17502001), 10 ng/ml of recombinant human neuregulin (Recombinant Human NRG1 Isoform SMDF; R & D Systems), 2 μm forskolin (Millipore Sigma, catalog number F3917), and 5 ng/ml of fibroblast growth factor (Gibco/ThermoFisher, catalog number PHG6015). Mouse MD-Schwann were originated in-house (35). Absence of merlin in mouse and human MD-Schwann cells was routinely tested by Western blotting for merlin expression (1:2,000, Cell Signaling Technology, catalog number 12888, RRID:AB_2650551).

### Human and mouse Schwann cells survival assay

Human and mouse Schwann cells were seeded at a density of 4 × 10^4^ cells per well in a 96-well plate and incubated for 48 or 96 h in the absence and presence of l-NAME (1, 2.5, and 5 mm, Millipore Sigma, catalog number N5751), FeTCPP (10 and 50 μm, Advance Biochemicals, catalog number POR0013), and urate (50 and 100 μm, Millipore Sigma, catalog number U2875). At the indicated times, the cells were washed twice with PBS and incubated for 20 min in 100 μl of 0.2% crystal violet and 20% methanol in PBS per well. Wells were washed with distilled water before adding 200 μl of solubilization solution (1.0% SDS) per well. Absorbance was measured at 595 nm and cell survival was expressed as percentage of untreated cells (% control).

### Subcellular fractionation

Cell homogenates were obtained as previously described (29, 62), with modifications. Human and mouse Schwann cells were disrupted in ice-cold MT buffer (0.3 m mannitol, 10 mm HEPES, pH 7.4) using a blunt 26-gauge needle. The disrupted cells were centrifuged at 1,000 × *g* for 10 min at 4 °C. To obtain the mitochondrial and cytosolic fractions, the cell homogenates were centrifuged at 12,000 × *g* for 10 min at 4 °C. The supernatant (cytosolic fraction) was centrifuged for an additional 20 min at 12,000 × *g*, 4 °C. The pellet (mitochondrial fraction) was resuspended in ice-cold MT buffer and centrifuged for an additional 10 min at 12,000 × *g*, 4 °C.

### Oxygen consumption rate

The OCR was measured in adherent human MD- and WT-Schwann cells using an XF24 Extracellular Flux Analyzer (Agilent Technologies, Santa Clara, CA), as previously described (29). When indicated, cells were treated in the presence and absence of 100 μm urate for 30 h, seeded in a XF24-well cell culture microplate (Agilent Technologies) at a density of 4 × 10^4^ cells per well, and incubated for an additional 18 h at 37 °C in 5% CO_2_. Then, media was replaced by seahorse basal medium supplemented with 25 mm glucose and 4 mm l-glutamine, and the cells were incubated for an additional hour. The oxygen consumption rate was determined under basal conditions and after the sequential addition of previously titrated: oligomycin (1 μm), carbonyl cyanide *p-*trifluoromethoxyphenylhydrazone (FCCP, 2 μm, Millipore Sigma, catalog number C2920), and antimycin A (10 μm, Millipore Sigma, catalog number A8674). The oxygen consumption rate data from each well was normalized to protein levels in the same well, and the nonmitochondrial respiration after addition of antimycin A was subtracted from all measurements. For complex I, II + III, and IV activities, cells were seeded at a density of 6 × 10^4^ cells per well and the activity measured as previously described, with modifications (29). Briefly, the activities were measured after the addition of Seahorse XF Plasma Membrane Permeabilizer, as recommended by the manufacturer (Agilent Technologies, catalog number 102504–100) together with ADP (1 mm, Millipore Sigma, catalog number A2754) and the corresponding substrate, followed by addition of appropriate inhibitors. Complex I and II + III activity were measured sequentially by addition of pyruvate (10 mm) and malate (1 mm), followed by the addition of rotenone (2 μm), then succinate (10 mm) followed by antimycin A (10 μm, Millipore Sigma). For complex IV activity, *N*,*N*,*N*′,*N*′-tetramethyl-*p*-phenylenediamine (TMPD, 0.5 mm, Millipore Sigma, catalog number T7394) and ascorbate (2 mm) were added together with antimycin A (10 μm), followed by azide (20 mm). Complex I activity in the presence of rotenone, complex II + III in the presence of antimycin A, and complex IV in the presence of azide were subtracted from all respective measurements and the activity expressed as inhibitor-sensitive complex activity.

### Glycolysis stress test

The extracellular acidification rate was measured in adherent human MD- and WT-HSC using the XF Glycolysis Stress Test kit (Agilent Technologies, catalog number 103020-100) according to the manufacturer's instructions. The cells were incubated in the presence and absence of 100 μm urate, as detailed for the oxygen consumption rate measurements, and seeded at a density of 6 × 10^4^ cells per well. To measure the extracellular acidification rate, the Seahorse base medium was supplemented with 1 mm glutamine, followed by sequential addition of 10 mm glucose, 1 μm oligomycin, and 50 mm 2-deoxyglucose to inhibit glycolysis.

### Glutamine dependence

To determine the percentage of glutamine dependence, human MD- and WT-HSC were incubated in the presence and absence of 100 μm urate, as described above, and seeded at a density of 6 × 10^4^ cells per well. The oxygen consumption rate was measured after the sequential addition of BPTES (3 μm) and UK5099 + Etomoxir (2 and 4 μm, respectively) using the XF Mito Fuel Flex Test kit (Agilent Technologies, catalog number 103260–100) according to the manufacturer's instructions.

### Quantitative IR Western blot analysis

IR Western blots were performed as previously described (28, 29, 36, 69). Briefly, 25–50 μg of total cell homogenates were loaded in 10, 12, or 15% gels followed by SDS-PAGE. Proteins were transferred to a polyvinylidene difluoride low fluorescence background membrane (Millipore Sigma), blocked using Odyssey Blocking Buffer (Li-Cor Biosciences, Lincoln, NE, catalog number 927-40000), and incubated with the indicated primary antibodies. IRDdye secondary goat antibodies (Li-Cor Biosciences), anti-mouse (680RD, catalog number 925-68070), and anti-rabbit (800CW, - 925-32211) were used at a 1:20,000 dilution. All Western blots were visualized and the bands quantified using the Odyssey System (Li-Cor Biosciences). After the densitometry analysis of the bands, treatments were normalized to the corresponding control samples, which were assigned as 1 in arbitrary units.

### Antibodies

All antibodies were used according to the manufacturer's instructions. VDAC (1:1,000, Cell Signaling Technology, catalog number 4866, RRID:AB_2272627); Hsp60 (1:1,000, Cell Signaling Technology, catalog number 12165, RRID:AB_2636980); complex IV (1;1,000, Cell Signaling Technology, catalog number 4850, RRID:AB_2085424); complex I (1:5,000, ThermoFisher Scientific, catalog number 459100, RRID:AB_2532223), cytochrome *c* (1:1,000, Cell Signaling Technology, catalog number 4280S, RRID:AB_10695410); pyruvate dehydrogenase (1:1,000, Cell Signaling Technology, catalog number 3205S, RRID:AB_2162926); SDHA (1:1,000, Cell Signaling Technology, catalog number 11998); Hsp90 (1:2,000, Santa Cruz Biotechnology, catalog number SC-7947); β-actin (1:30,000, Cell Signaling Technology, catalog number 3700, RRID:AB_2242334); α-tubulin (1:30,000, Cell Signaling Technology, catalog number 3873, RRID:AB_1904178); merlin (1:2,000, Cell Signaling Technology, catalog number 12888, RRID:AB_2650551); MnSOD (1:1,000, Millipore Sigma, catalog number 06-984); nNOS (Santa Cruz Biotechnology, catalog number sc-648, RRID:AB_630935); iNOS (Santa Cruz Biotechnology, catalog number sc-650, RRID:AB_631831); and eNOS (1:1,000, BD Biosciences, catalog number 610296, RRID:AB_397690). The mouse mAb against nitrated Hsp90 (1:2,000) was developed and characterized in-house (28), as well as the rabbit polyclonal antibody against nitrotyrosine (1:2,000) (70).

### Mitochondrial content using MitoTracker Green FM

Human Schwann cells mitochondrial content was assayed by measuring MitoTracker Green FM fluorescence in live cells according to the manufacturer's instructions (200 nm, Molecular Probes/ThermoFisher Scientific, catalog number M7514) using a Synergy H1 multi-mode reader (BioTek, Winooski, VT). Cells were washed with DPBS once before measuring MitoTracker Green fluorescence.

### Mitochondrial count and area per cell

Human Schwann cell mitochondrial count and area per cell was assayed in live cells after incubation with previously titrated MitoTracker Red FM (350 nm) for 30 min according to the manufacturer's instructions (Molecular Probes/ThermoFisher Scientific, catalog number M22425). When indicated, cells were incubated in the presence or absence of 100 μm urate for 48 h and washed with DPBS before the addition of the mitochondrial probe. Cell culture plates were then placed on a Zeiss widefield fluorescence microscope with full incubation. Representative fields were imaged using a ×40/0.6 LD Plan Neofluar objective and a ×63/1.4 Plan Apochromat objective, a HXP 120C light source set to 50% light with Filter Set 43 HE, and a Hamamatsu ORCA-R2 camera. Volocity 6.3 software was used to deconvolve images (using Iterative Restoration to a confidence limit of 99% and an appropriate calculated PSF) and then identify individual mitochondria. Mitochondria area/cell and number/cell was measured per field of view.

### Mitochondrial membrane potential of human Schwann cells

Human MD- and WT-Schwann cells were plated in a 96-well plate (4 × 10^4^ cells per well) and incubated for 24 h at 37 °C in 5% CO_2_/air before adding the probe JC-1 (5 μm, Molecular Probes/ThermoFisher Scientific, catalog number T3168). The cells were then incubated for an extra 30 min followed by a wash with DPBS. Fluorescence was measured at 560 excitation/590 emission (*red*) and 485 excitation/530 emission (*green*) and the data expressed as the ratio of red/green signals. When indicated, 10 μm FCCP was added to the cells together with the JC-1. To measure mitochondrial membrane potential after urate treatments, cells were treated in the presence and absence of 100 μm urate for 30 h, seeded in a 96-well plate at a density of 4 × 10^4^ cells per well, and incubated for an additional 18 h before assessing JC-1 fluorescence.

### Mitochondrial complex activities in disrupted mitochondria

The measurement of complex I, II + III, and IV activities was performed in disrupted mitochondria as previously described (29, 62). The mitochondrial fraction was subjected to 3 cycles of freeze/thawing prior to measurements. Complex I activity was measured at 340 nm by the rotenone (10 μm)-sensitive reduction of 50 μm ubiquinone-1 (Millipore Sigma, catalog number C7956) in the presence of 1 mm potassium cyanide (Millipore Sigma, catalog number 60178), 3 mg/ml of fatty acid-free BSA (Millipore Sigma, catalog number A7030), and 200 μm NADH as electron donor (Millipore Sigma, catalog number N4505). Activity of complexes II + III was determined by the antimycin A (20 μm, Millipore Sigma)-sensitive reduction of 50 μm cytochrome *c* (Millipore Sigma, catalog number C7752) at 550 nm in the presence of 1 mm potassium cyanide (Millipore Sigma, catalog number 60178) and 10 mm succinate (Millipore Sigma, catalog number S3674). The potassium cyanide (1 mm)-sensitive activity of complex IV was determined by monitoring cytochrome *c* oxidation at 550 nm. The activities were expressed as nanomole/min/mg of protein.

### Statistical analysis

For statistical analyses, a test for outliers was conducted on the datasets and assessment of normality was carried out using Prism (GraphPad Software Inc., San Diego, CA). For those populations following a normal distribution, Student's *t* test was performed when comparing two groups and one-way ANOVA followed by Bonferroni multiple comparison test when comparing more than two groups. For populations not following a normal distribution, Wilcoxon test or Kruskal-Wallis nonparametric tests followed by Dunn's multiple comparison test were used. All tests were performed using the program Prism.

## Author contributions

J. C. P., S. K. P., O. D. V. C., M. A. F., M. O., N. A. S., and M. C. F. formal analysis; J. C. P., S. K. P., O. D. V. C., M. A. F., M. O., N. A. S., and M. C. F. investigation; J. C. P., C. F.-V., and M. C. F. writing-original draft; J. C. P., M. A. K., C. F.-V., and M. C. F. writing-review and editing; M. A. K., C. F.-V., and M. C. F. resources; C. F.-V. and M. C. F. supervision; C. F.-V. and M. C. F. funding acquisition; C. F.-V. and M. C. F. methodology; C. F.-V. and M. C. F. project administration; M. C. F. conceptualization.

## Footnotes

4 The abbreviations used are:

NF2: neurofibromatosis type 2
Hsp: heat shock protein
MD: merlin-deficient
OCR: oxygen consumption rate
VS: vestibular schwannomas
MnSOD: manganese superoxide dismutase
eNOS: neuronal nitric-oxide synthase
iNOS: inducible nitric-oxide synthase
eNOS: endothelial nitric-oxide synthase
VDAC: voltage-dependent anion channel
BPTES: bis-2-(5-phenylacetamido-1,3,4-thiadiazol-2-yl-ethyl sulfide
FCCP: *p-*trifluoromethoxyphenylhydrazone
TMPD: *N*,*N*,*N*′,*N*′-tetramethyl-*p*-phenylenediamine
DPBS: Dulbecco's phosphate-buffered saline
ANOVA: analysis of variance
HSC: human Schwann cell
MSC: mouse Schwann cell
PD: pyruvate dehydrogenase
FeTCPP: Fe(III)-meso-Tetra(4-carboxyphenyl)porphine chloride
l-NAME: *N*γ-*nitro-l-arginine methyl ester*.
